# The microvascular effects of insulin resistance and diabetes on cardiac structure, function, and perfusion: a cardiovascular magnetic resonance study

**DOI:** 10.1093/ehjci/jeu142

**Published:** 2014-08-12

**Authors:** Abdulghani M. Larghat, Peter P. Swoboda, John D. Biglands, Mark T. Kearney, John P. Greenwood, Sven Plein

**Affiliations:** 1 Multidisciplinary Cardiovascular Research Centre & Leeds Institute of Genetics, Health and Therapeutics, University of Leeds, Leeds LS2 9JT, UK; 2 Department of Cardiology and Internal Medicine, Tripoli Medical Centre, Faculty of Medicine, University of Tripoli, Tripoli, Libya; 3 Division of Medical Physics, University of Leeds, Leeds, UK

**Keywords:** Diabetes, Prediabetes, Cardiovascular magnetic resonance, Tagging, Strain, Myocardial perfusion reserve

## Abstract

**Aims:**

Type 2 diabetes mellitus is an independent risk factor for the development of heart failure. To better understand the mechanism by which this occurs, we investigated cardiac structure, function, and perfusion in patients with and without diabetes.

**Methods and results:**

Sixty-five patients with no stenosis >30% on invasive coronary angiography were categorized into diabetes (19) and non-diabetes (46) which was further categorized into prediabetes (30) and controls (16) according to the American Diabetes Association guidelines. Each patient underwent comprehensive cardiovascular magnetic resonance assessment. Left-ventricular (LV) mass, relative wall mass (RWM), Lagrangian circumferential strain, LV torsion, and myocardial perfusion reserve (MPR) were calculated. LV mass was higher in diabetics than non-diabetics (112.8 ± 39.7 vs. 91.5 ± 21.3 g, *P* = 0.01) and in diabetics than prediabetics (112.8 ± 39.7 vs. 90.3 ± 18.7 g, *P* = 0.02). LV torsion angle was higher in diabetics than non-diabetics (9.65 ± 1.90 vs. 8.59 ± 1.91°, *P* = 0.047), and MPR was lower in diabetics than non-diabetics (2.10 ± 0.76 vs. 2.84 ± 1.25 mL/g/min, *P* = 0.01). There was significant correlation between MPR and early diastolic strain rate (*r* = −0.310, *P* = 0.01) and LV torsion (*r* = −0.306, *P* = 0.01). In multivariable linear regression analysis, non-diabetics waist–hip ratio, but not body mass index, had a significant association with RWM (Beta = 0.34, *P* = 0.02).

**Conclusion:**

Patients with diabetes have increased LV mass, LV torsion, and decreased MPR. There is a significant association between decreased MPR and increased LV torsion suggesting a possible mechanistic link between microvascular disease and cardiac dysfunction in diabetes.

## Introduction

Cardiovascular disease is a major cause of morbidity and mortality in patients with diabetes mellitus.^1^ Type 2 diabetes has long been recognized as a major risk factor for the development of coronary artery disease (CAD),^2^ but diabetes and insulin resistance are now also recognized as independent risk factors for the development of heart failure.^3,4^ In one observational study, 33% of men and 45% of women with diabetes developed heart failure over 5.5-year follow-up.^5^ The risk of developing heart failure was independent of age, gender, CAD, or hypertension. Once established, heart failure in patients with diabetes is associated with worse clinical outcomes, independent of CAD.^6^ Observational studies have demonstrated that patients with systolic heart failure and diabetes have a mortality that is almost doubled compared with their normoglycaemic counterparts.^7,8^

Several mechanisms have been proposed for the association between diabetes and heart failure including endothelial dysfunction, abnormal calcium handling, myocardial fibrosis, and inappropriate activation of the renin–angiotensin–aldosterone system.^9,10^ Observational research has shown that diabetes is associated with an increase in left-ventricular (LV) mass^11^ and impaired diastolic function.^12^ Over time, these structural and functional changes lead to impaired systolic function and the clinical syndrome of heart failure.^13^

Cardiovascular magnetic resonance (CMR) offers a unique opportunity to assess these changes in cardiac structure and function as well as changes in myocardial perfusion in a single examination.

In this study, we undertook a comprehensive assessment of cardiac structure, function, and perfusion by CMR in patients with diabetes, prediabetes, and normal insulin sensitivity, in whom significant CAD was excluded by coronary angiography. We hypothesized that patients with diabetes have increased LV mass, abnormal strain patterns, and impaired myocardial perfusion and that patients with prediabetes would have similar, albeit less severe, changes.

## Methods

### Selection of patients and recruitment

We prospectively screened 399 consecutive patients with non-obstructive (no stenosis >30% luminal narrowing by visual analysis) CAD on routine coronary angiography typically clinically indicated for the investigation of chest pain at our tertiary cardiology centre. Patients with a history of previous myocardial infarction, coronary revascularization or other significant heart disease, contraindications to CMR or adenosine, and known claustrophobia were excluded. Of the remaining patients, 72 agreed to participate in the study. The study was approved by the local Ethics Committee. All patients gave fully informed written consent.

### Patient classification

Smoking history, clinic non-invasive blood pressure, lipid profile, and drug history were recorded. In line with current American Diabetes Association (ADA) guidelines,^14^ diabetes mellitus was defined as fasting glucose ≥7 mmol/L, HbA1c ≥6.5%, or a past history of diabetes. Duration of diabetes was reported by the patients. Those that did not meet this definition were defined as non-diabetes. Prediabetes was defined as fasting glucose 5.6–6.0 mmol/L or HbA1c 5.7–6.4%. Those that did not meet the definition of diabetes or prediabetes were defined as controls. Hypertension was defined as clinic SBP >140 mmHg (the level at which treatment is recommended in current ADA guidelines). Hypercholesterolaemia was defined as current use of HMG-CoA reductase inhibitors (statins) or low-density lipoprotein (LDL) >100 mg/dL (the level at which statins are recommended in ADA guidelines). All subjects had their height, weight, hip circumference, and waist circumference measured. The homeostatic model assessment of insulin resistance (HOMA-IR) was calculated using the following formula: serum fasting glucose (µU/mL) × serum fasting insulin (mg/dL)/405.^15^ In diabetic patients, this was only calculated for those not taking exogenous insulin. Waist–hip ratio (WHR), body mass index (BMI), and Mosteller body surface area (BSA) were calculated.

### Blood and urine sampling

Urine was tested for albumin–creatinine ratio. Blood samples were obtained from participants after 8 h of fasting and tested for glucose, insulin, total cholesterol, LDL cholesterol, high-density lipoprotein cholesterol, serum urea and electrolytes, and glycosylated haemoglobin (HbA1c).

### CMR protocol

CMR was performed on a 1.5 T whole-body magnetic resonance scanner (Intera, Philips Medical Systems, Best, The Netherlands) using vectorcardiographic gating and a 5-element cardiac phased-array receiver coil. Data were acquired during breath holding at end expiration. From scout CMR images, the LV long and short axes were determined. Then, tagged CMR images were acquired at the apex, mid-ventricle, and base [complementary spatial modulation of magnetization method (CSPAMM) using multishot echo planar imaging, flip angle sweep applied to the radiofrequency excitation pulses of subsequent cardiac phases, two orthogonal line tags acquired per slice in a 14 s breath hold, typical FOV 300 mm, matrix 128 × 128, slice thickness 10 mm, tag separation 8 mm, 18 phases, temporal resolution 30 ms, and typical repetition time (TR)/echo time (TE)/flip angle 30 ms/6 ms/25°]. Slices were positioned using the highly reproducible ‘3 of 5 technique’.^16^

Next, myocardial perfusion CMR was planned in the mid-LV short axis orientation with a saturation recovery fast gradient echo method accelerated by two-fold sensitivity encoding (SENSE), TR/TE/flip angle 2.7/1.0/15°, typical field of view 380 × 380 mm, image matrix 160 × 160, in plane spatial resolution 2.4 × 2.4 mm, slice thickness 10 mm, 60 dynamics, preparation pulse delay (to middle of k-space) 150 ms, and shot duration 130 ms.^17^ For perfusion acquisition, a contrast dose of 0.05 mmol/kg gadopentetate dimeglumine (Magnevist, Bayer-Schering Pharma, Berlin, Germany) was administered at a rate of 5 mL/s followed by a 20 mL saline flush. Breath holding was carried out during the first pass of contrast agent. The same mid-myocardial section was imaged twice–once in mid-systole and end-diastole by choosing two appropriate trigger delays from the cine images. A stress perfusion scan was performed during maximal vasodilatation, stimulated by intravenous infusion of adenosine at a dose of 140 µg/min/kg for 4 min.

Then, a retrospectively triggered, balanced steady-state free precession (SSFP) short-axis cine stack covering the entire LV was acquired (TR/TE/flip angle 3.3/1.64/60°, typical FOV 380 × 380 mm, matrix 192 × 256, slice thickness 10 mm, slice gap: 0–1 mm, 12 slices, temporal resolution 40 ms, 20 phases).

After a 15 min delay from stress perfusion imaging, a rest perfusion scan was performed in identical locations and using the same approach as the stress acquisition followed by an additional top-up bolus of 0.1 mmol/kg Gd-DTPA.

### LV volumes and mass quantification

Using dedicated image analysis software (Q Mass 6.1.6, Medis, Leiden University, Leiden, The Netherlands), the epicardial and endocardial borders were traced off line on the LV cine stack.^18^ End-diastolic, end-systolic (ES) LV volumes, stroke volume (SV), ejection fraction (EF), and LV mass including papillary muscles were calculated. Relative wall mass (RWM) was calculated by dividing LV mass by LV end-diastolic volume (EDV).^19^ Left atrial volume was calculated from the SSFP 4- and 2-chamber cine at end-systole using the following formula:^20^

$$\text{LA}\;\text{volume}=\frac{8\times 4\;\text{chamber}\;\text{area}\times 2\;\text{chamber}\;\text{area}}{3\times \pi \times \text{length}\;\text{of}\;\text{the}\;\text{left}\;\text{atria}}.$$

### Quantitative myocardial blood flow estimation

**Figure 1:**
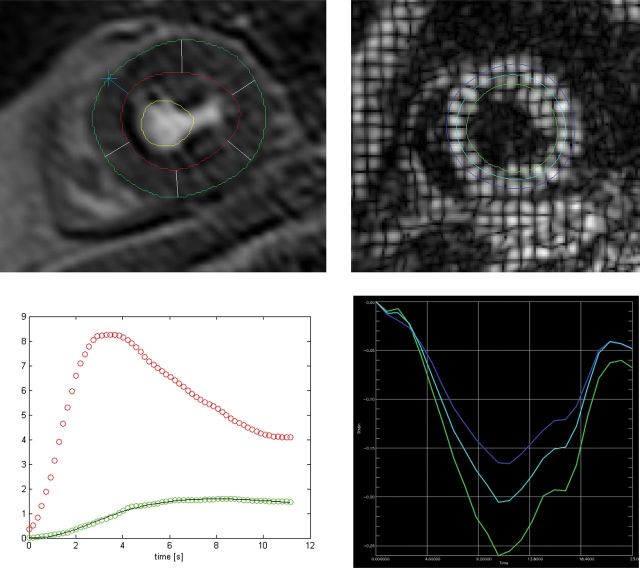
Mid-myocardial stress perfusion contours (top left) and blood flow after Fermi-constrained deconvolution (bottom left) with MBF (green) and AIF (red). Mid-myocardial CSPAMM tagging showing contours (top right) and Lagrangian circumferential strain over time (bottom right) with each colour representing a different layer: epicardium (blue), mid-myocardium (cyan), and endocardium (green)

Analysis was performed with the same dedicated software package. Endocardial and epicardial contours were outlined on one representative dynamic perfusion frame with the optimum blood-to-myocardial contrast, with papillary muscles excluded, and copied to all other dynamic frames (*Figure 1*). The position of individual contours was then manually corrected to account for any respiratory motion. In order to obtain an arterial input function (AIF), another region of interest was drawn inside the LV blood pool. This method has previously been shown to be highly reproducible.^21^ Myocardial blood flow (MBF) was measured in the systolic phase where the myocardium is thicker and easier to contour and has previously been shown to be as accurate as diastolic MBF measurement.^22^ AIF was measured in the diastolic phase when there was the largest area of blood pool to contour. Signal intensity vs. time profiles were then generated for the mid-LV myocardial slice as a whole without dividing into segments and for the LV blood pool. Signal intensity vs. time data generated in MASS were analysed with MATLAB7 R2009b, (The MathWorks Inc., Natick, USA). Fermi-constrained deconvolution was used to generate estimates of absolute MBF in mL/g/min.^23^ The myocardial perfusion reserve (MPR) was calculated by dividing hyperaemic (stress) by baseline (rest) MBF.

### CSPAMM analysis

Analysis was performed for the entire myocardial slice from each acquisition with a dedicated tagging analysis software package using HARP analysis (TagTrack, GyroTools, Zurich, CH, Switzerland). Endocardial and epicardial contours were drawn by a semi-automated process for each slice, and a mid-myocardial contour was automatically calculated between the endocardial and epicardial contours (*Figure 1*). The software then tracked the contours throughout all phases of the cardiac cycle. Circumferential Lagrangian strain and strain rate were measured at the mid-ventricular level, which has previously been shown to be the most reproducible.^16^ Early diastolic strain rate was defined as peak rate in the first four phases after end-systole. LV twist was calculated by subtracting the basal rotation from the apical rotation. LV torsion was calculated by the following formula:^24^

$$\begin{array}{l} \text{Torsion shear angle}=\\ \;\frac{\left(\text{Apical rotation}-\text{Basal rotation})\times (\text{Apical radius}+\text{Basal radius}\right)}{\text{Length}\times 2}. \end{array}$$

### Statistical analysis

Statistical analysis was performed using IBM SPSS^®^ Statistics 19.0. Continuous variables were expressed as means ± standard deviation (SD). Means of the diabetes and non-diabetes groups were compared using an unpaired two-tailed *t*-test with assumed equal variance. To compare the diabetes, prediabetes, and control groups, analysis of variance with *post hoc* Tukey correction was used. A *P*-value of <0.05 was considered statistically significant. Kolmogorov–Smirnov test was used to establish that MPR was not normally distributed. Correlations between MPR and strain measurements were assessed by Spearman’s test. Univariable analyses were performed to identify predictors of increased LV mass index (LVMI), RWM, and LV torsion and reduced MPR. Variables with a probability value of <0.1 in the univariable analysis were included in a stepwise multivariable analysis, based on a linear regression model. Error bars for mean values denote 95% confidence interval.

## Results

Of the 72 recruited patients, three could not complete the scan because of claustrophobia. Two patients in the diabetes group had tagging data of poor quality that could not be interpreted. In two patients, one of the perfusion data sets was of insufficient quality for quantitative analysis (excessive through plane motion). These four patients were excluded from the final analysis. Of the 65 patients with completed scans, 19 were classed as diabetic and 46 non-diabetics (of whom 30 were defined as prediabetic). The mean duration of diabetes was 7.3 ± 9.0 years.

Patient characteristics of the study groups are shown in *Table 1*. The proportion of males (68 vs. 41%, *P* = 0.048) was higher in the diabetics than non-diabetics. Serum creatinine (93.7 ± 8.7 vs. 85.4 ± 6.4 µmol/L, *P* = 0.01) and WHR (0.95 ± 0.11 vs. 0.90 ± 0.09, *P* = 0.046) were higher in diabetics than non-diabetics. There was no significant difference between diabetics and non-diabetics for age, BMI, hypertension, hypercholesterolaemia, albumin–creatinine ratio, or smoking history. HOMA-IR was significantly higher in diabetics (not taking exogenous insulin) than in non-diabetics (10.0 ± 9.1 vs. 2.5 ± 1.5, *P* < 0.001), but there was no significant difference between prediabetics and controls.

**Table 1:** Patient characteristics with total numbers in each group

	Diabetes	Non-diabetes	Prediabetes	Control
Number	19	46	30	16
Age^a^	59 ± 6	57 ± 7	57 ± 8	57 ± 7
Male	13 (68)^†^	19 (41)	13 (43)	6 (38)
Hypertension	7 (37)	16 (35)	11 (37)	5 (31)
Hypercholesterolaemia	16 (84)	38 (82)	26(87)	12 (75)
Current smoking	2 (9)	6 (13)	4 (13)	2 (13)
BMI (kg/m^2^)^a^	30.81 ± 4.6	29 ± 4.9	30.1 ± 5.0	27.7 ± 4.5
WHR^a^	0.95 ± 0.11^†^	0.90 ± 0.09	0.91 ± 0.09	0.88 ± 0.09
Clinic SBP (mmHg)^a^	134.3 ± 14.43	132.8 ± 15.3	131.6 ± 15.0	135.1 ± 15.9
Clinic DBP (mmHg)^a^	76.4 ± 9.7	77.2 ± 9.5	76.9 ± 8.7	77.8 ± 11.1
LDL cholesterol (mmol/L)	75.7 ± 16.6^†^	116.2 ± 18.9	115.8 ± 42.1	116.6 ± 34.0
HbA1c (%)^a^	7.7 ± 1.6^†^	5.77 ± 0.29	5.9 ± 0.2	5.5 ± 0.2
Serum creatinine (µmol/L)^a^	93.7 ± 8.7^†^	85.4 ± 6.4	86.6 ± 14.2	83.1 ± 10.6
Urine ACR (mg/mmol)^a^	3.8 ± 8.7	1.2 ± 1.83	1.2 ± 1.9	1.1 ± 1.6
HOMA-IR^a^	10.0 ± 9.1^†^	2.5 ± 1.5	2.7 ± 1.4	2.2 ± 1.7
Medication				
ACE-i/ARB	14 (74)^†^	14 (30)	10 (33)	4 (25)
Beta-blocker	11(58)	17 (37)	14 (47)	3 (19)
Calcium channel blocker	4(21)	11 (24)	7 (23)	4 (25)
Thiazide diuretic	4 (21)	3 (7)	3 (10)	0 (0)
HMG-CoA reductase inhibitor (statin)	14 (61)^†^	17 (37)	10 (33)	7 (44)
Metformin	9 (47)^†^	0 (0)	0 (0)	0 (0)
Insulin	5 (26)^†^	0 (0)	0 (0)	0 (0)

Percentage of nominal values in parentheses.

^a^Data are mean ± SD.

^†^*P* < 0.05 when compared with non-diabetes.

When diabetics, prediabetics, and controls were compared, serum creatinine was higher in diabetics than controls (93.7 ± 8.7 vs. 83.1 ± 10.6 µmol/L, *P* = 0.03). There were no other differences in patient characteristics.

### LV structure

LV mass (112.8 ± 39.7 vs. 91.5 ± 21.3 g, *P* = 0.01), EDV (171.0 ± 43.0 vs. 151.1 ± 26.2 mL *P* = 0.03), and SV (95.9 ± 25.7 vs. 86.6 ± 12.2 mL *P* = 0.05) were higher in diabetics than non-diabetics. There were no significant differences in LVMI, RWM, LA area, or LA area indexed to BSA (*Table 2*).

**Table 2:** CMR measured mean and SD LV structure, function, and perfusion

	Diabetes		Non-diabetes		Prediabetes		Control	
	Mean	SD	Mean	SD	Mean	SD	Mean	SD
LV mass (g)	112.8^†^	39.7	91.5	21.3	90.3	18.7	93.7	26.0
LVMI Mosteller (g/m^2^)	52.5	12.8	47.7	8.9	46.8	7.0	49.4	11.7
EDV (mL)	171.0^†^	43.0	151.1	26.2	152.0	25.2	149.4	29.0
ESV volume (mL)	73.5	22.9	64.5	17.8	64.3	16.6	64.8	20.4
SV	95.9*	25.7	86.6	12.2	87.7	12.7	84.6	11.3
RWM (g/mL)	0.65	0.14	0.61	0.10	0.60	0.09	0.63	0.11
LA volumes (mL)	100.0	27.3	91.3	17.2	89.6	19.0	94.3	13.4
LA volume index to BSA (mL/m^2^)	47.2	10.0	47.7	7.7	46.4	8.7	50.0	5.2
EF (%)	57.5	5.5	57.8	5.2	58.0	5.1	57.4	5.6
Peak circumferential strain	−0.18	0.03	−0.18	0.03	−0.18	0.03	−0.18	0.03
Peak systolic strain rate (S^−1^)	−0.90	0.22	−0.91	0.17	−0.93	0.19	−0.88	0.13
Peak early diastolic strain rate (S^−1^)	0.52*	0.25	0.40	0.20	0.41	0.17	0.40	0.24
LV twist (degrees)	10.88^†^	2.61	9.37	0.41	9.60	2.43	8.93	1.70
LV torsion (degrees)	9.65*	1.90	8.59	1.91	8.69	2.11	8.41	1.51
Stress MBF (mL/g/min)	3.39*	0.85	4.05	1.35	4.10	1.42	3.96	0.31
Rest MBF (mL/g/min)	1.81	0.80	1.54	0.52	1.54	0.57	1.54	0.11
MPR (mL/g/min)	2.10^†^	0.76	2.84	1.25	2.88	1.34	2.76	0.27

^†^*P* < 0.05 when compared with non-diabetes.

*P = 0.05 when compared with non-diabetes.

On comparing diabetics, prediabetics, and controls, LV mass was higher in diabetics than prediabetics (112.8 ± 39.7 vs. 90.3 ± 18.7 g, *P* = 0.02).

### LV function

There was no significant difference in EF, peak Lagrangian circumferential strain, or systolic strain rate between diabetics and non-diabetics (*Table 2*). LV twist (10.88 ± 2.6 vs. 9.37 ± 0.41°, *P* = 0.02) and shear torsion angle (9.65 ± 1.90 vs. 8.59 ± 1.91°, *P* = 0.047) were significantly higher in diabetics than non-diabetics. Early diastolic strain rate (0.52 ± 0.25 vs. 0.40 ± 0.20 S^−1^, *P* = 0.051) was higher in diabetics than non-diabetics bordering on significance.

Diabetics had LV twist that was significantly greater than prediabetics (10.88 ± 2.61 vs. 9.60 ± 2.43°, *P* = 0.04).

### Myocardial blood flow

**Figure 2:**
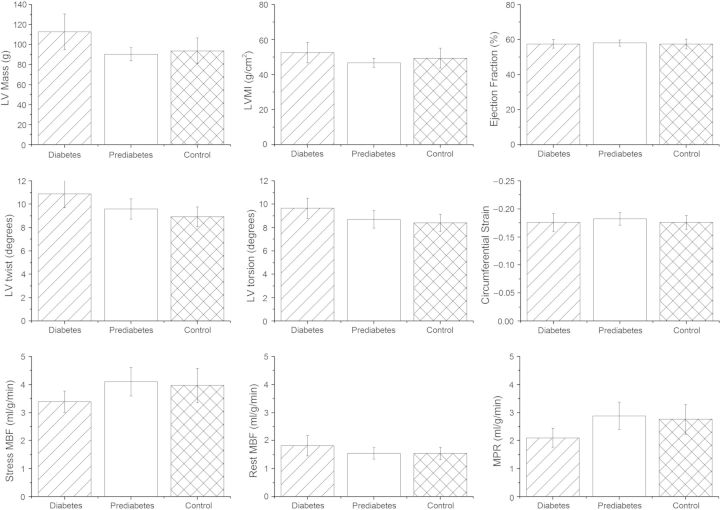
Mean and 95% confidence intervals of LV mass, LVMI, and EF (top); LV twist, LV torsion, and circumferential strain (middle); and stress MBF, rest MBF, and MPR (below). Within each graph diabetes is on the left, prediabetes in the middle, and controls on the right

There was no significant difference in MBF at stress or at rest between diabetics and non-diabetics (*Figure 2*). However, MPR was significantly decreased (2.10 ± 0.76 vs. 2.84 ± 1.25 mL/g/min, *P* = 0.01) in diabetics compared with non-diabetics.

MPR was decreased in diabetics compared with prediabetics of borderline significance (2.10 ± 0.76 vs. 2.88 ± 1.34 mL/g/min, *P* = 0.05). There was no difference in MPR between prediabetics and controls.

Calculation of Spearman’s correlation coefficient revealed a modest significant correlation between MPR and early diastolic strain rate (*r* = −0.310, *P* = 0.01) and LV torsion (*r* = −0.306, *P* = 0.01).

### Multivariable linear regression analysis

In the multivariable linear regression model of all patients (*Table 3*), only male sex was associated with increased LVMI (Beta = 0.57, *P* < 0.001). RWM had a significant association with WHR only (Beta = 0.44, *P* < 0.001). The only factor significantly associated with increased LV torsion shear angle was a history of diabetes (Beta = 0.25, *P* = 0.047). On univariable regression analysis, age, sex, and diabetes had a correlation with decreased MPR (*P* < 0.1) and were included in the multivariable analysis. In the multivariate analysis, diabetes (Beta = −0.27, *P* = 0.03) and age (Beta = −0.27, *P* = 0.02) had a significant association with decreased MPR.

**Table 3:** Univariable and multivariable linear regression analysis of all patients

	LVMI (g/m^2^)		RWM (g/mL)		LV torsion (degrees)		MPR (mL/g/min)	
	Univariable *P*-value	Multivariable *P*-value	Univariable *P*-value	Multivariable *P*-value	Univariable *P*-value	Multivariable *P*-value	Univariable *P*-value	Multivariable *P*-value
Age	0.44		0.51		0.08	0.19	0.01	0.02*
Sex	<0.001	<0.001*	<0.001	0.052	0.39		0.099	0.24
Hypertension	0.44		0.64		0.13		0.30	
Diabetes	0.09	0.47	0.11		0.05	0.047*	0.01	0.03*
Prediabetes	0.09	0.15	0.10		0.43		0.13	
Microalbuminuria	0.68		0.53		0.42		0.99	
BMI	0.66		0.13		0.86		0.57	
WHR	0.007	0.87	<0.001	<0.001*	0.57		0.87	
Current smoking	0.14		0.73		0.19		0.93	
Hypercholesterolaemia	0.58		0.93		0.82		0.30	

Factors with *P* < 0.1 in the univariable analysis were included in the multivariable analysis.

*Significant correlation *P* < 0.05.

In the multivariable linear regression model of only non-diabetic patients (*Table 4*), only male sex was associated with increased LVMI (Beta = 0.50, *P* < 0.001). RWM had a significant association with WHR (Beta = 0.34, *P* = 0.02). None of the risk factors had a significant association with LV torsion. MPR only had a significant association with male sex (Beta = 0.49, *P* = 0.001).

**Table 4:** Univariable and multivariable linear regression analysis in non-diabetic patients

	LVMI (g/m^2^)		RWM (g/mL)		LV Torsion (degrees)		MPR (mL/g/min)	
	Univariable *P*-value	Multivariable *P*-value	Univariable *P*-value	Multivariable *P*-value	Univariable *P*-value	Multivariable *P*-value	Univariable *P*-value	Multivariable *P*-value
Age	0.28		0.83		0.12		0.12	
Sex	<0.001	<0.001*	0.020	0.16	0.20		0.026	0.026*
Hypertension	0.88		0.64		0.36		0.35	
Microalbuminuria	0.39		0.35		0.22		0.86	
BMI	0.33		0.86		0.82		0.94	
WHR	0.09	0.68	0.023	0.023*	0.55		0.83	
Current smoking	0.09	0.06	0.63		0.31		0.64	
Hypercholesterolaemia	0.06	0.06	0.43		0.67		0.37	
HOMA-IR	0.14		0.95		0.97		0.43	

Factors with *P* < 0.1 in the univariable analysis were included in the multivariable analysis.

*Significant correlation *P* < 0.05.

## Discussion

We have demonstrated that Type 2 diabetes mellitus, when compared with both non-diabetes and prediabetes, is associated with increased LV mass and LV torsion and decreased MPR.

Data from the large observational Multi-Ethnic Study of Atherosclerosis study^11^ have previously demonstrated that diabetes is associated with an increase in LV mass. After regression analysis, diabetes was associated with a 3.5 g greater LV mass (95% confidence interval 1.2–5.8 g). The findings of the present study corroborate these findings and also demonstrate that diabetic subjects have an increase in LV mass compared with subjects with prediabetes.

Previous observational studies have demonstrated that insulin resistance and prediabetic states are associated with concentric LV remodelling^19^ and abnormal LV function.^25,26^ In our study, patients with prediabetics had LVMI and RWM similar to controls; however, in the multivariable linear regression analysis of all subjects and non-diabetics RWM (a marker of LV remodelling) had a significant correlation with WHR but not BMI or HOMA-IR. The role of visceral adipose tissue in cardiac remodelling and dysfunction is increasingly being recognized,^27^ and our finding of a correlation between WHR and LV remodelling independent of diabetes status or HOMA-IR adds further evidence to this hypothesis.

Further, we have demonstrated that diabetics have increased LV twist and torsion despite unchanged circumferential strain. This finding has previously been reported in patients with Type 1 diabetes mellitus and was attributed to subendocardial myocardial dysfunction secondary to small vessel disease.^28^ Our results and our finding of a negative correlation between MPR and LV torsion suggest that this hypothesis is also applicable in Type 2 diabetes. It is plausible that abnormal myocardial perfusion in Type 2 diabetes contributes to the progressive changes in cardiac strain and function reported in this study and in previous studies.^12,29^ The hypothesis that impaired MPR leads to cardiac dysfunction raises the possibility of future therapeutic trials of pharmacological agents that increase coronary microvascular function to prevent heart failure. One recent study in which patients with diabetes were randomized to receive the selective phosphodiesterase inhibitor sildenafil or placebo^30^ reported that sildenafil reversed LV remodelling as well as decreasing LV torsion compared with placebo. The possible mechanistic link between microvascular disease and cardiac dysfunction does suggest a need for further trials into drugs that vasodilate the coronary microvasculature.

It has been reported in previous CMR-based research that diabetic patients when compared with controls have increased LV torsion^12^ and decreased MPR.^31^ In both of these studies, the diabetic patients had significantly higher blood pressure (BP) than the controls. It has previously been reported that hypertension leads to increased torsional shear angle, decreased circumferential strain,^32^ and decreased MPR.^33,34^ In our study, there was no significant difference in clinic BP between the study groups making the reported changes likely due to diabetic status of the patients.

We had hypothesized that patients with prediabetes would also show abnormalities in LV structure, function, and perfusion. The techniques employed in this study did not demonstrate any abnormalities in prediabetes. Our findings have also confirmed MPR measurements by positron emission tomography^35^ where MPR was also reduced in diabetics but not prediabetics.^36^ Other CMR techniques such as T1 mapping^37,38^ or measuring aortic distensibility^39,40^ have been demonstrated to show changes in diabetes and may be more sensitive to detect subclinical cardiac changes in prediabetes.

### Limitations

The three patient groups had similar characteristics, but there were more men and higher creatinine in the diabetic group than non-diabetic group. We attempted to control for this retrospectively by indexing results to BSA where appropriate and by conducting a multivariable linear regression analysis. The correlations reported are modest which reflects the fact that multiple factors influence ventricular structure, function, and perfusion.

Diabetics also had significantly higher use of angiotensin-converting-enzyme (ACE) inhibitors and statins than non-diabetic patients. This may have influenced our results, but any effects would be expected to improve the studied parameters in patients taking ACE inhibitors or statins.

We have only carried out CSPAMM and MPR analysis at the mid-ventricular short axis level because this has previously been demonstrated to be the most reproducible level for analysis of both of these techniques.^16,21^ We have not reported the segmental analysis of CSPAMM or MPR because we are investigating a diffuse process that affects the whole myocardium. With regard to MPR, our aim was to optimize image acquisition and our study aims to examine ubiquitous changes in the myocardium rather than to detect coronary disease (which was excluded at patient inclusion). For this purpose, single slice coverage was considered sufficient.

Patients were recruited at the time of coronary angiography, typically for investigation of chest pain. It is possible that these patients by nature of their symptoms are not representative of all diabetic or non-diabetic patients.

## Conclusions

Patients with diabetes, without CAD, have increased LV mass, LVMI, and LV torsion and decreased MPR when compared with non-diabetic patients. There is a significant association between decreased MPR and increased LV torsion suggesting a possible mechanistic link between microvascular disease and cardiac dysfunction in diabetes.

**Conflict of interest:** None declared.

## Funding

Libyan Ministry of Higher Education & Scientific Research, Tripoli, Libya to A.M.L. and British Heart Foundation to S.P. (FS/10/62/28409). S.P. and J.P.G. received unrestricted educational grants from Philips Healthcare. Funding to pay the Open Access publication charges for this article was provided by The University of Leeds.
